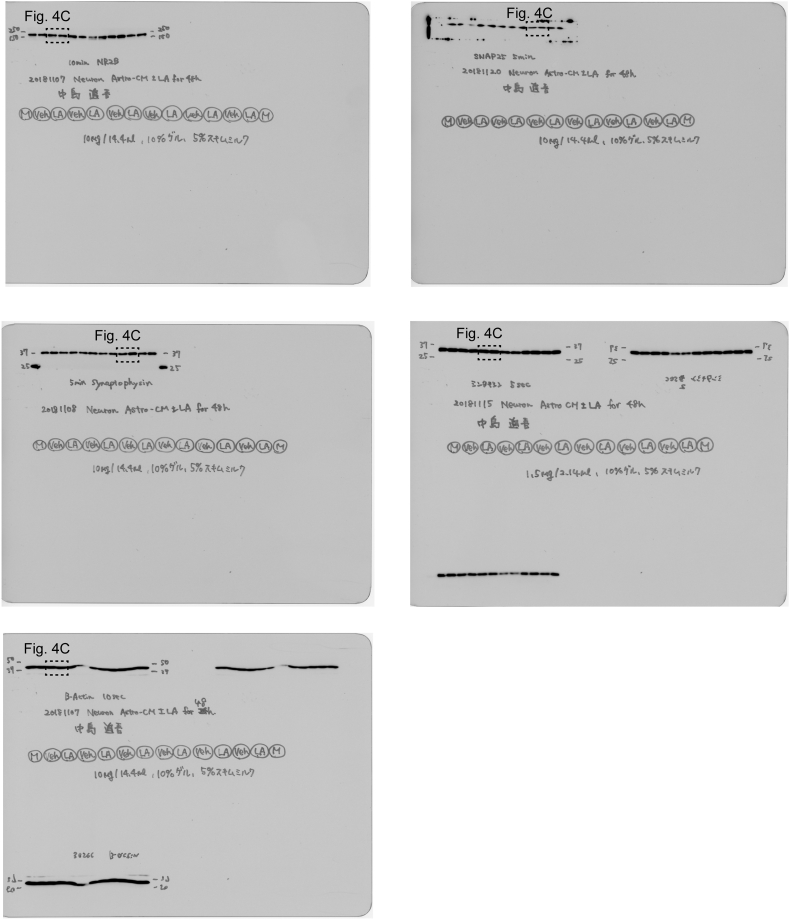# Lauric acid promotes neuronal maturation mediated by astrocytes in primary cortical cultures

**DOI:** 10.1016/j.heliyon.2020.e03892

**Published:** 2020-05-11

**Authors:** Shingo Nakajima, Hiroshi Kunugi

**Affiliations:** a Department of Mental Disorder Research, National Institute of Neuroscience, National Center of Neurology and Psychiatry (NCNP), Tokyo, Japan; b Department of Psychiatry, Teikyo University School of Medicine, Tokyo, Japan

**Keywords:** Neuroscience, Cellular neuroscience, Molecular neuroscience, Cell culture, Physiology, Lauric acid, Neurotrophic factor, Cytokine, Neuron-astrocyte interaction, Extracellular signal-regulated kinase

## Abstract

Previous studies have suggested the potential efficacy of middle chain fatty acids (MCFAs) in the treatment of mood disorders and cognitive dysfunction. MCFAs are metabolized to ketone bodies in astrocytes; however, their effects on neuronal development including neurotrophic factor level are not well-understood. In the present study, we examined the effect of MCFAs on the mRNA expression of growth factors and cytokines in primary cultures of cortical astrocytes. The effect of MCFAs on neuron-astrocyte interaction in neuronal maturation was also determined using co-culture and astrocyte-conditioned medium. Lauric acid (LA) typically increased the mRNA expression of glial-derived neurotrophic factor (*Gdnf*), interleukin-6 (*Il6*), and C–C motif chemokine 2 (*Ccl2*) in astrocytes. LA-induced phosphorylation of extracellular signal-regulated kinase contributed to these changes. In primary cultures of cortical neurons containing astrocytes, LA enhanced the presynaptic protein levels. Astrocyte-conditioned medium after LA treatment also enhanced the presynaptic protein levels in the cortical neuron cultures. These results suggest that LA increase the mRNA expression of GDNF and cytokines in astrocytes, and thereby, enhances the presynaptic maturation.

## 1 Introduction

The intake of middle chain fatty acids (MCFAs) as a ketogenic diet has been suggested to have a therapeutic effect on mood disorders and cognitive dysfunctions [1, 2, 3]. MCFAs such as capric acid, caprylic acid, and lauric acid (LA) are catabolized to ketone bodies, such as acetate and β-hydroxy butyrate (BHB) [4, 5]. Interestingly, MCFAs not only provide ketone bodies but also protect cortical neurons against amyloid β-induced toxicity [6]. Coconut oil, which contains a high amont of LA [7], prevents stress-induced depressive- and anxiety-like behaviors in rodents [8]. Although the beneficial effect of MCFAs is gradually being recognized, their effect on neuronal functions has not been well-understood.

Neurotrophic factors such as glial-derived neurotrophic factor (GDNF) and brain-derived neurotrophic factor (BDNF) are essential for maintenance and development of the central nervous systems [9, 10, 11]. Cytokines are well known regulators of the immune system response to pathogen-associated molecules such as bacterial lipopolysaccharide (LPS) [12]. They contribute to the maintenance of neuro-immune interactions [13, 14]. Celluler communication between neurons and astrocytes is important for neuronal functions such as neurotransmitter release, protection of activity-induced lipotoxicity, and the maintenance of neuronal environment [15, 16]. Astrocytes produce growth factors and cytokines in response to extracellular stimuli [17, 18]. Saturated fatty acids and *n*-3 polyunsaturated fatty acids induce the production of cytokines in astrocytes and neurotrophic factors in neurons, respectively [19, 20]. However, the effect of these fatty acids on neural function with respect to neuron-astrocyte communication is still unclear.

In the present study, we tested the effect of ketone bodies produced due to the intake of MCFAs on the production of growth factors and cytokines at transcriptional level in cultured cortical astrocytes. We also examined the signaling pathways such as extracellular signal-regulated kinase (ERK) and nuclear factor kappa B (NFκB) in relation to the mRNA expression of growth factors and cytokines. The effect of MCFAs on neuron-astrocyte interaction in neuronal maturation was determined using co-culture system and astrocyte-conditioned medium.

## 2 Materials and methods

### 2.1 Reagents

BHB and sodium salts of capric acid, caprylic acid, LA, myristic acid, palmitic acid, and stearic acid were purchased from Tokyo Chemical Industry Co., Ltd. (Tokyo, Japan). Anti-ERK, anti-phosphorylated ERK (pERK), anti-Akt, anti-pAkt, anti-NFκB, anti-pNFκB, and U0126 were purchased from Cell Signaling Technology Inc. (Danvers, MA, USA). Anti- N-methyl-D-aspartate receptor type 2B (NR2B), anti-syntaxin, anti-β-actin, LPS (from *Escherichia coli* O111:B4), and poly-ethylenimine (PEI) were purchased from Sigma-Aldrich (St. Louis, MO, USA). Cytarabine (AraC) was purchased from FUJIFILM-Wako Pure Chemical Corp. (Osaka, Japan). Anti-synaptophysin (Boehringer Mannheim GmbH, Mannheim, Germany) and anti-synaptosomal nerve-associated protein 25 (SNAP25) (Synaptic Systems, Germany) were also used in the study.

### 2.2 Cell culture

Primary cultures of cortical neurons and astrocytes were prepared from Wistar rats at postnatal day 1–2 according to previous reports with minor modifications [21, 22]. Briefly, the dissected cortex was treated with papain (9 unit/mL) and DNase I (15 unit/mL) in phosphate-buffered saline for 20 min at 37 °C. The cell suspension was mixed and then filtered using a 70 μm cell strainer.

Mixed glial cells were cultured in a 75 cm^2^ flask until they reached semi-confluency (for 7–9 days). To obtain the astrocyte cultures, oligodendrocyte precursor cells and microglia were detached by tapping every 2–3 days. Astrocytes were detached by trypsinization. The cells were then seeded to 24-well or 3.5 cm-dish at a density of 2.5 × 10^4^ cells/cm^2^.

Primary cultures of neurons were seeded in a PEI-coated 3.5 cm-dish with a neuronal medium (DMEM/F12 containing 5% fetal bovine serum, 5% horse serum, 100 μg/mL streptomycin, and 100 units/mL penicillin) at a density of 5 × 10^5^ cells/cm^2^. To inhibit glial proliferation, primary neurons were cultured in the presence of 2 μM AraC from 1 day *in vitro* (DIV 1).

The experiments were approved by the Ethics Review Committee for Animal Experimentation of the National Institute of Neuroscience, Japan (approval number; 2017019).

### 2.3 Treatment of fatty acids and conditioned medium

Stock solution of each sodium fatty acid salt was prepared in distilled water at 100 mM by ultrasonication with heating. At day 6 after re-seeding, astrocytes were treated to several types of fatty acids or BHB at concentrations and durations indicated in the figure legends. The mitogen-activated protein kinase kinase (MEK) inhibitor (U0126) was pre-treated for 15 min prior to the application of LA. For preparation of a conditioned medium, astrocyte-conditioned medium was replaced with a neuronal medium and LA was applied for 24 h. Cultured cortical neurons at DIV 6 were treated with LA or were replaced to the astrocyte-conditioned medium for 48 h in the presence or absence of AraC.

### 2.4 Real-time PCR

Total RNA from astrocytes was extracted using the TRI Reagent® (Molecular Research Center Inc., Cincinnati, OH, USA) according to the manufacturer's protocol. Equivalent amount of total RNA (1 μg) was provided for complementary DNA synthesis using SuperScript® VIRO™ cDNA synthesis kit (Thermo Fisher Scientific Inc. Waltham, MA, USA). The amplification of synthesized cDNA with SYBR green (TOYOBO CO., Ltd. Osaka, Japan) and each gene-specific primer set (Supplemental Table 1) was performed using StepOnePlus™ real-time PCR systems (Thermo Fisher Scientific Inc.). The expression level of each target gene was normalized by the expression level of glyceraldehyde-3-phosphate dehydrogenase. The mRNA expression was shown as a relative level compared to control.

### 2.5 Western blotting

Lysis buffer containing 1% SDS, 10 mM Tris-HCl, 10 mM Na_4_P_2_O_7_, 10 mM NaF, 5 mM EDTA, 2 mM NaVO_4_ and 1 mM PMSF was used to collect the cell lysate. Concentration of each protein was measured with Pierce® BCA protein assay (Thermo scientific, IL, USA). Equivalent amount of protein was used in the SDS-PAGE, and the separated protein on the acrylamide gel was transferred to PVDF membrane (Millipore, MA, USA). After blocking with 5% skim milk for 1 h, the primary antibody was applied to the membrane overnight. After washing with tris-based saline, the incubation of secondary antibody for mouse (Jackson ImmunoResearch Europe Ltd., Suffolk, UK) or rabbit (Rockland Immunochemicals, Inc., Gilbertsville, PA, USA) was performed. The chemiluminescence was detected using Immunostar® reagent (FUJIFILM-Wako Pure Chemical Corp., Osaka, Japan) and ECL films (GE healthcare UK Ltd., Buckinghamshire, UK). The optical density of protein expression was measured using CS Analyzer version 3.00.1011 (ATTO Co. Tokyo, Japan).

### 2.6 Statistical analysis

All data are expressed as means ± SEM. Data were analyzed with Student's *t*-test and One-way ANOVA with Dunnett's multiple comparison test or Tukey's post-hoc test using Prism 8 software (GraphPad software, San Diego, CA, USA). Probability values less than 5% were considered statistically significant.

## 3 Results

### 3.1 LA highly enhanced Gdnf mRNA expression in cultured cortical astrocytes

**Figure 1:**
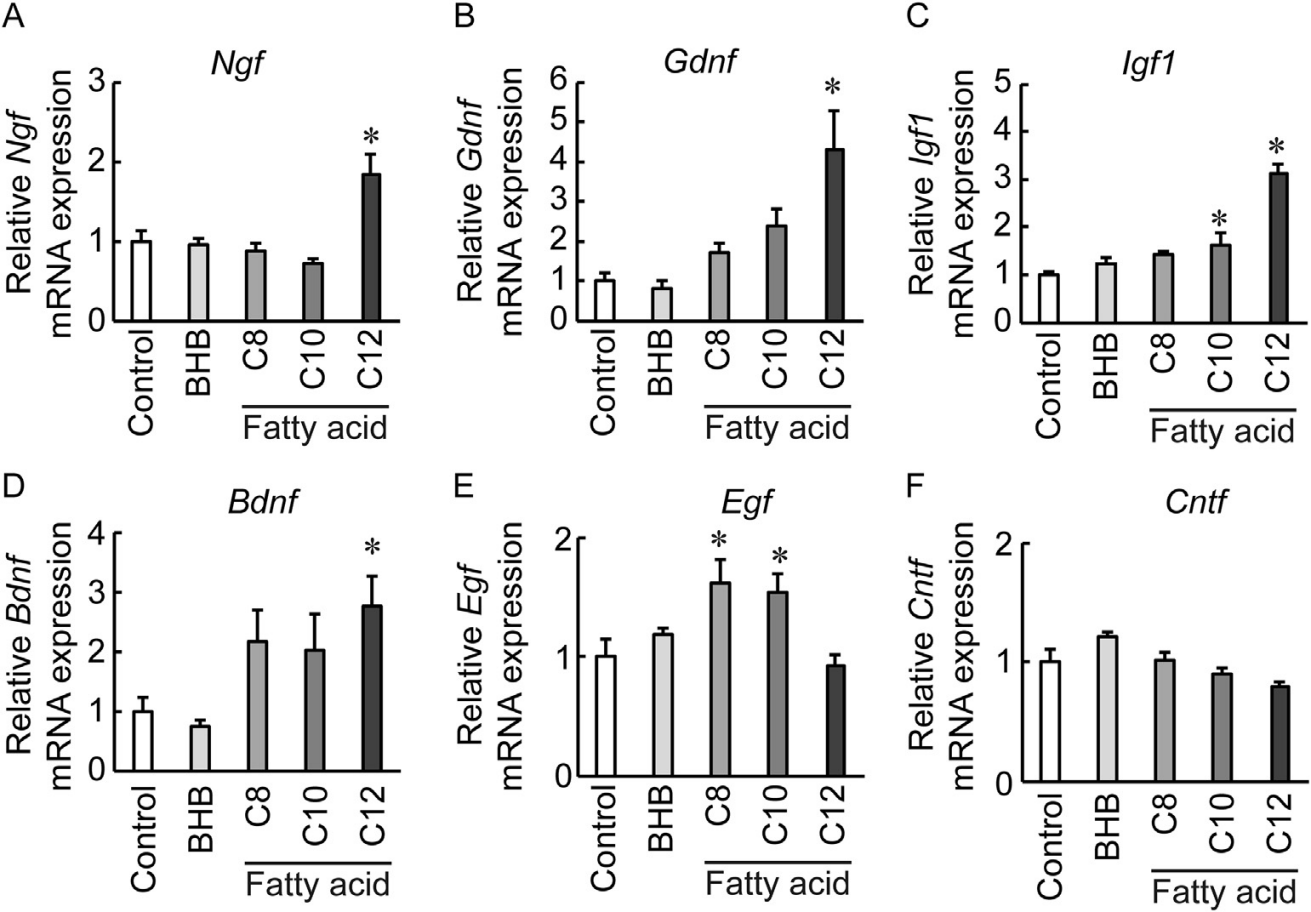
Effect of β-hydroxy butyrate (BHB) and middle chain fatty acids on the mRNA expression of growth factors in primary culture of astrocytes. Relative mRNA expressions of (A) nerve growth factor (*Ngf*), (B) glial cell-derived neurotrophic factor (*Gdnf*), (C) Insulin-like growth factor (*Igf1*), (D) brain-derived neurotrophic factor (*Bdnf*), (E) epidermal growth factor (*Egf*), and (F) ciliary neurotrophic factor (*Cntf*) at 3 h after 300 μM of capric acid (C8), caprylic acid (C10), or LA (C12) treatment in primary culture of astrocytes (n = 4–5) are shown. The expression of target genes was normalized with the internal standard gene (glyceraldehyde-3-phosphate dehydrogenase). Values represent mean ± SEM. ∗*P* < 0.05 vs. control (Dunnett's test).

We compared the effect of BHB and MCFAs on the mRNA expression of growth factors in astrocytes. Although BHB had no significant effect on the expression of the examined growth factor, LA significantly increased the mRNA expression of nerve growth factor *(Ngf)*, *Gdnf*, insulin-like growth factor-1 (*Igf1*), and *Bdnf* (Figure 1A, 1B, 1C, and 1D). On the other hand, capric acid and caprylic acid significantly increased the mRNA expression of epidermal growth factor (*Egf*) mRNA (Figure 1E). The expression of ciliary neurotrophic factor (*Cntf*) was not significantly changed by BHB or the MCFAs (Figure 1F).

### 3.2 Different expression pattern between lauric acid and LPS

**Figure 2:**
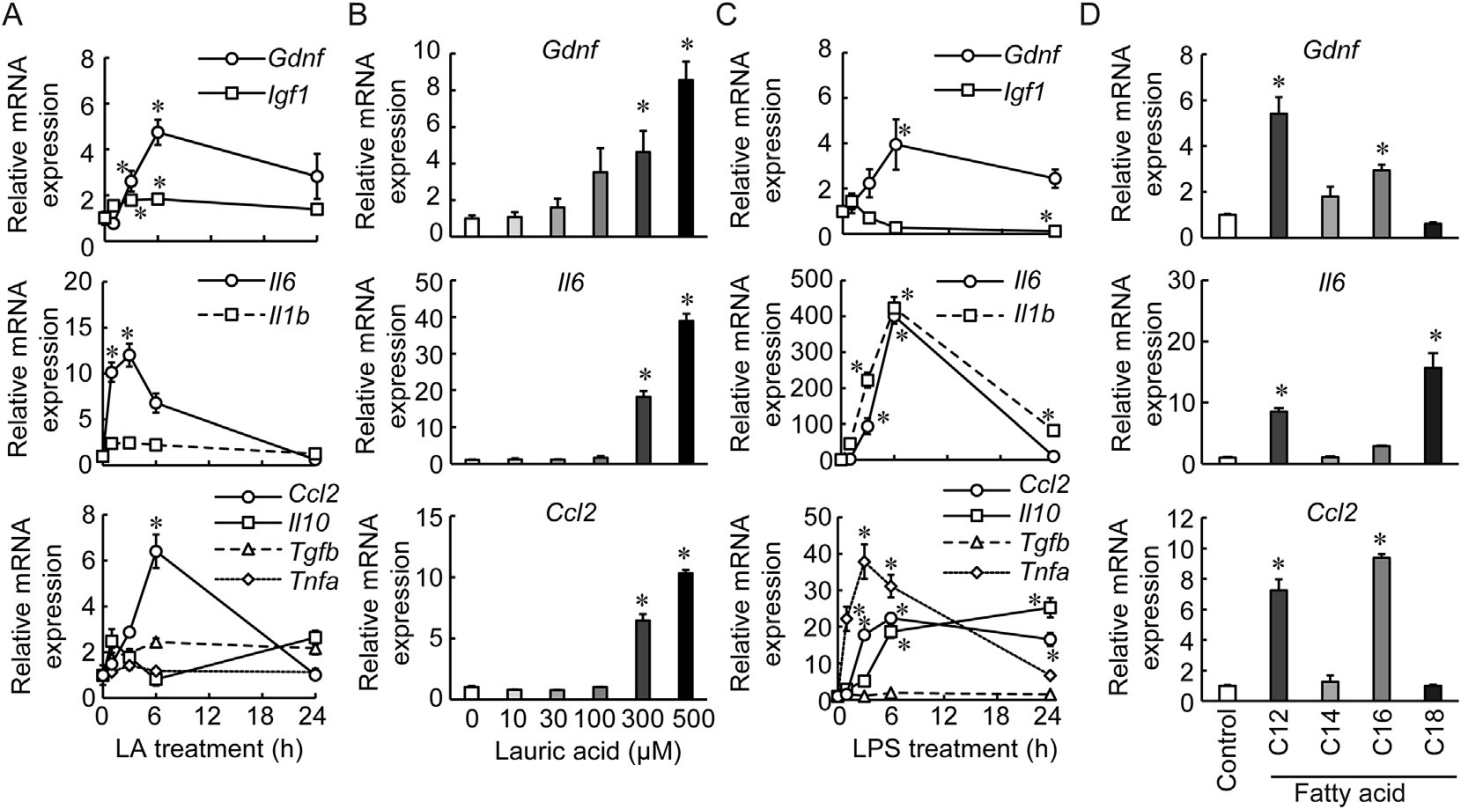
The mRNA expression patterns of growth factors and cytokines after treatment with lauric acid (LA), lipopolysaccharide (LPS), or saturated long chain fatty acids in primary culture of astrocytes. (A) Time course (300 μM LA) and (B) dose response (at 6 h) after LA treatment in the cultured astrocytes (n = 4) with respect to glial cell-derived neurotrophic factor (*Gdnf*), insulin-like growth factor (*Igf1*), interleukin 6 (*Il6*), interleukin 1b (*Il1b*), C–C motif chemokine 2 (*Ccl2*), interleukin 10 (*Il10*), transforming growth factor β (Tgfb), and tumor necrosis factor α (Tnfa). (C) Time course of 10 ng/ml LPS treatment on the mRNA expression in astrocytes (n = 4). (D) *Gdnf*, *Il6*, and *Ccl2* mRNA expression after LA (C12), myristic acid (C14), palmitic acid (C16), or stearic acid (C18) treatment (300 μM, 6 h) (n = 4–5). The expression of target genes was normalized with the internal standard gene. Values represent mean ± SEM. ∗*P* < 0.05 vs. control (Dunnett's test).

Increased cytokine secretion induced by saturated fatty acids in astrocytes has been demonstrated [19]. Therefore, we examined the dose and time-dependent effect of LA on the mRNA expressions of the highly related growth factors (*Gdnf* and *Igf1*) as well as several cytokines such as interleukin 1b (*Il1b*), interleukin 6 (*Il6*), tumor necrosis factor α (*Tnfa*) and C–C motif chemokine 2 (*Ccl2*). At 6 h after the LA treatment, *Gdnf*, *Il6*, and *Ccl2* mRNA expression was significantly higher compared to those after no treatment (Figure 2A). LA increased the mRNA expressions of these factors in a dose-dependent manner (Figure 2B). On the other hand, LPS increased the expression of various cytokines (*Il1b*, *Il6*, *Tnfa*, *Ccl2*, and *Interlaukin 10*) to a far greater extent than LA. Higher *Gdnf* and lower *Igf1* mRNA expressions were observed after the LPS treatment (Figure 2C). Palmitic acid significantly increased the *Gdnf* and *Ccl2* mRNA expression, whereas stearic acid only increased only the *Il6* mRNA expression (Figure 2D). Together with the effect of LA, no dependency of carbon-chain length was observed on *Gdnf*, *Il6*, and *Ccl2* mRNA expression in astrocytes (Figure 2D).

### 3.3 Intracellular signaling after lauric acid treatment

**Figure 3:**
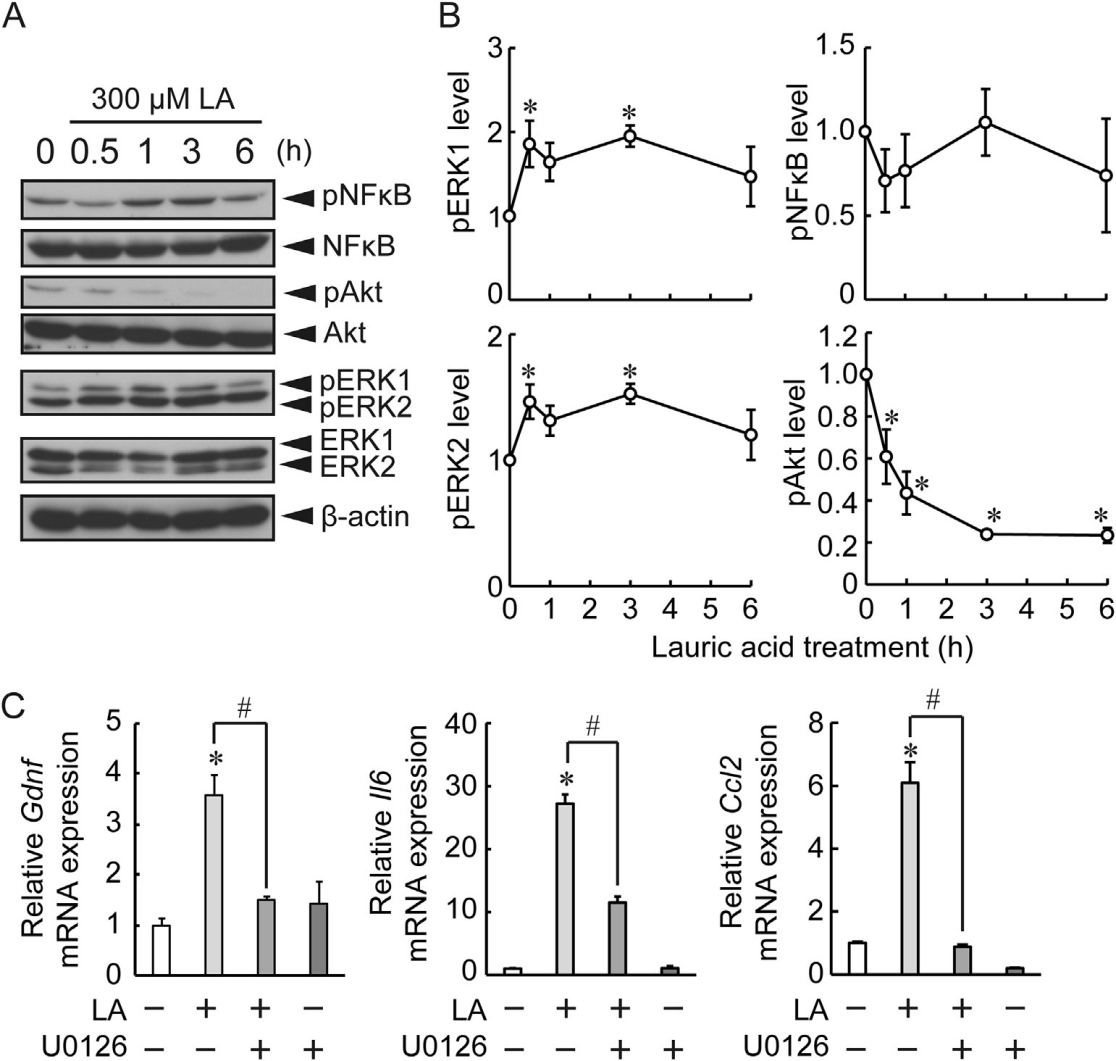
Contribution of extracellular signal-regulated kinase (ERK) to lauric acid (LA)-induced mRNA expression of growth factors and cytokines in primary cultured astrocytes. (A) Representative image of each signal protein after LA treatment. The original images are shown in supplementary material. (B) Relative phosphorylated levels of ERK, NFkB, and Akt after LA treatment (300 μM) compared to no-treatment (0 min) (n = 5). (C) *Gdnf*, *Il6*, and *Ccl2* mRNA expression at 3 h after LA treatment (300 μM) in the presence or absence of MEK inhibitor (U0126, 10 μM) (n = 4). The expression of target genes was normalized with the internal standard gene. Values represent mean ± SEM. ∗*P* < 0.05 vs. control, ^#^*P* < 0.05, Dunnett's test or Tukey's post hoc test.

Expression of growth factors is mediated by intracellular signaling molecules such as ERK and Akt [17], whereas expression of cytokines is regulated by NFκB and ERK [18]. After LA treatment, pERK levels were significantly elevated compared to the basal levels (Figure 3B), while pAkt levels were significantly decreased (Figure 3B). No significant change was observed in the pNFκB levels (Figure 3B). To determine the contribution of increased pERK levels, we examined the effect of the MEK inhibitor (U0126) on LA-induced mRNA expressions. LA-induced *Gdnf* and *Ccl2* mRNA expressions were abolished by the U0126 (Figure 3C). Increased *Il6* mRNA expression after LA treatment was partially but significantly decreased by the U0126 (Figure 3C).

### 3.4 Lauric acid induced neural maturation mediated by astrocytes

**Figure 4:**
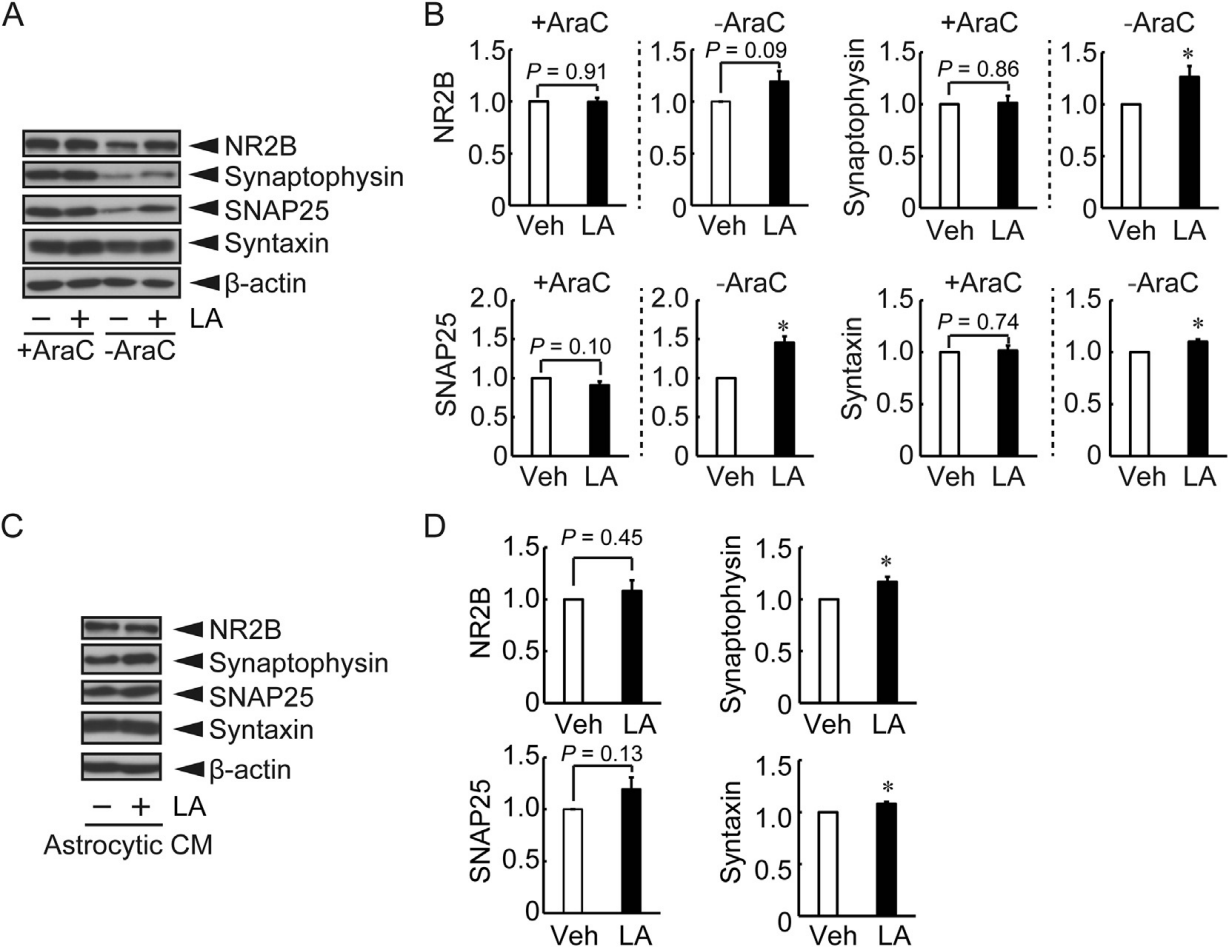
Pre-synaptic and post-synaptic protein levels after LA treatment with/without inhibition of glial proliferation by cytarabine (AraC) and conditioned medium from LA-treated astrocytes in primary neuron-rich cultures. (A) Representative image of each synaptic protein after 300 μM LA treatment for 48 h in the presence or absence of AraC (2 μM). The original images are shown in supplementary material. (B) Relative protein levels for NR2B, synaptophysin, SNAP25, and syntaxin after the LA treatment with/without AraC (n = 5). (C) Representative image of each synaptic protein level at 48 h after the exposure of conditioned medium from astrocytes in AraC-treated cultured cortical neurons. The original images are shown in supplementary material. (D) Relative protein levels for NR2B, synaptophysin, SNAP25, and syntaxin after exposure to vehicle (veh) or 300 μM LA treated-conditioned medium from astrocytes (n = 5–6). Values represent mean ± SEM. ∗*P* < 0.05 vs. control (Student's *t*-test).

GDNF, IL6, and CCL2 are known to induce neural maturation including axon branching and neurite outgrowth [10, 23, 24]. Therefore, we examined the effect of LA on astrocyte-mediated neuronal maturation. AraC is widely used to increase the neuronal population by inhibition of glial proliferation including that of astrocytes [25], which enables the examination of the contribution of astrocytes. Protein level of NR2B, a post-synaptic protein, was not significantly changed after LA treatment in the presence of AraC (highly neuronal culture) and in the absence of AraC (cultured cortical neurons containing astrocytes) (Figure 4A, B). On the other hand, LA treatment increased the pre-synaptic protein (synaptophysin, SNAP25, and syntaxin) levels in cultured cortical neurons containing astrocytes (Figure 4A, B).

We also tested the effect of conditioned medium from LA-treated astrocytes on these synaptic protein levels. The conditioned medium from LA-treated astrocytes had no effect on the NR2B levels, whereas significant increase in synaptophysin and syntaxin, and increased tendency of SNAP25 by the conditioned medium were observed (Figure 4C, D).

## 4 Discussion

Neuron-glial cell communication in lipid metabolism is necessary to regulate the neuronal functions such as neurotransmission and neural development [15]. It is well-known that astrocytes provide lactate and ketone bodies to neurons through the catabolism of MCFAs [4]. LA is metabolized to ketone bodies in an astrocyte cell line [5]. It has been demonstrated that fatty acids and ketone bodies regulate the production of neurotrophic factors and cytokines [19, 20, 26, 27]. In the present study, LA was found to increase the mRNA expression of *Gdnf*, *Il6*, and *Ccl2* in cultured cortical astrocytes. Although the dose of LA was slightly high in the present study, MCFAs and oleic acids have been tested at 300 μM without cytotoxicity in primary cultured astrocytes [4, 28]. Further, another paper has showed LA and oleic acid at high-dose (maximum 500 μM) are not toxic in neuroblastoma and glioblastoma cell lines [29]. It is well known that saturated fatty acids activate the toll-like receptor 4 to induce the cytokine expression in immune cells [30]. Further, saturated catty acids (LA, palmitate, and sterate) enhance cytokines (TNFα, IL1β and IL6) secretion from primary cultured astrocytes [19]. In contrast to previous report, the mRNA expression of major inflammatory cytokines (Tnfa and Il1b) was not increased after LA treatment, while they were increased after treatment with LPS in our astrocytic cultures. One of the explanation for this discrepancy is high-glucose enhances the expression of cytokines in astrocytes [31]. However, mRNA of Gdnf, Il6, and Ccl2 were increased by LA application at high-glucose condition, suggesting that these changes are independent to extracellular glucose levels. Importantly, LA activated the mitogenic pathway (through pERK) but not the inflammatory pathway (through pNFκB) to induce these mRNA expressions in astrocytes. It has been reported that LA attenuates the inflammatory response induced by LPS in primary microglia or microglial cell line BV-2 in the dependent of GPR40 [32]. LA activated GPR40 and TLR4 as same as longer saturated fatty acids [33, 34], however; longer saturated fatty acids are component of cellular structure such as cell membrane and lipid storage as demonstrated in lipidomic analysis [28], which may explain the specificity of LA in the astrocytic mRNA production. In addition, shorter (C8 and C10) carbon chain fatty acids are easier to be metabolized to ketone bodies [35]. These results suggest that LA may contribute to regulate neural immune response compared to other saturated fatty acids.

GDNF, IL6, and CCL2 have a potential to promote axon branching and neurite outgrowth [10, 23, 24], which leading us to examine the effect of LA on neurons through the astrocyte. Our neuron-astrocyte co-culture system and treatment with astrocyte-conditioned medium showed that LA-induced presynaptic maturation was mediated by astrocytes. Synaptophysin, SNAP25, and syntaxin are major protein of the vesicle component in related to exocytosis of neurotransmitter [36, 37], which are well correlated to neurotransmitter release in our neuronal culture [21, 38]. Interestingly, presynaptic ATP supply for basal and high-demand transmission is differentially regulated by itself or astrocytes [39]. Mitochondria are located predominantly in neuronal somata and primary dendrites, whereas it has demonstrated low abundance of mitochondria and high abundance of ATP at presynaptic terminals [40]. Taken together with our result, LA is a possible nutrient to enhance the neuronal maturation through the promoting of presynaptic vesicle protein content; however, it has been undetermined what protein secreted from astrocytes by LA and its receptor in neurons, which means that it is required further study to identify the mediating factors that contribute to this presynaptic maturation between neurons-astrocytes.

MCFAs in triglycerides in a form of ketogenic diet could be one of the new treatment strategies for mood disorders [1]. Cognition and synaptic plasticity are enhanced by the supplementation of triglycerides containing capric and caprylic acids in rats [41]. Application of capric and caprylic acids-rich triglycerides improved cognitive function in elderly adults and in patients with mild-to-moderate Alzheimer's disease [2, 3]. In the present study, BHB, capric acid, and caprylic acid had lesser effects on the mRNA expression of neurotrophic factors compared to the effect of LA in astrocytes. However, capric acid and caprylic acid-increased *Egf* expression was not significantly enhanced by LA. These results suggest that the effects on the expression of neurotrophic factors may be different depending on the carbon chain length of the MCFAs. Because increased ketone bodies are not only BHB but also acetoacetate by MCFAs [2], it is required to examine the effect of acetoacetate on expression of neurotrophic factors to clarify the contribution of ketone bodies on the effect of MCFAs in future study. It is also possible that capric acid and caprylic acid act predominantly as an energy sources to maintain neuronal energetics. Importantly, coconut oil, which mainly contains LA [7], induces enhanced memory function and reduces the anxiety-like behavior though LA. However, it has lesser ability to produce astrocytic ketone bodies compared to caprylic acid [5, 8, 42]. Moreover, it has been demonstrated that coconut oil directly activates the neuronal ERK and Akt signaling to prevent amyloid β toxicity [6]. Our results also showed that LA had a unique characteristic to produce several growth factors when compared with the other MCFAs in cultured cortical astrocytes, suggesting that LA might show a unique potential for the treatment of psychiatric and neurodegenerative diseases.

In conclusion, the present study demonstrated that LA has a potential to enhance the mRNA expression of growth factors and cytokines such as *Gdnf*, *Il6*, and *Ccl2*. These increases were mediated by the ERK signaling. LA-induced neuronal maturation required the presence of astrocytes. Astrocyte-conditioned medium also increase the presynaptic protein levels. These results suggest that LA could be one of the lipid activator for the neuron-glial cell communication for neuronal development.

## Declarations

### Author contribution statement

Shingo Nakajima: Conceived and designed the experiments; Performed the experiments; Analyzed and interpreted the data; Contributed reagents, materials, analysis tools or data; Wrote the paper.

Hiroshi Kunugi: Conceived and designed the experiments; Wrote the paper.

### Funding statement

This work was supported by 10.13039/501100001691Japan Society for the Promotion of Science (JSPS) KAKENHI Grant Numbers 16K16589 (S. N.) and 19J40268 (S. N.), the Strategic Research Program for Brain Sciences from 10.13039/100009619Japan Agency for Medical Research and Development, 10.13039/100009619AMED Grant number 17dm0107100h0002 (H. K.), and a research grant from Ryoshoku, the 10.13039/501100008669Food Science Institute Foundation, Japan (H. K.).

### Competing interest statement

The authors declare no conflict of interest.

### Additional information

No additional information is available for this paper.

## Figures and Tables

**Supple Ref to Fig3A:**
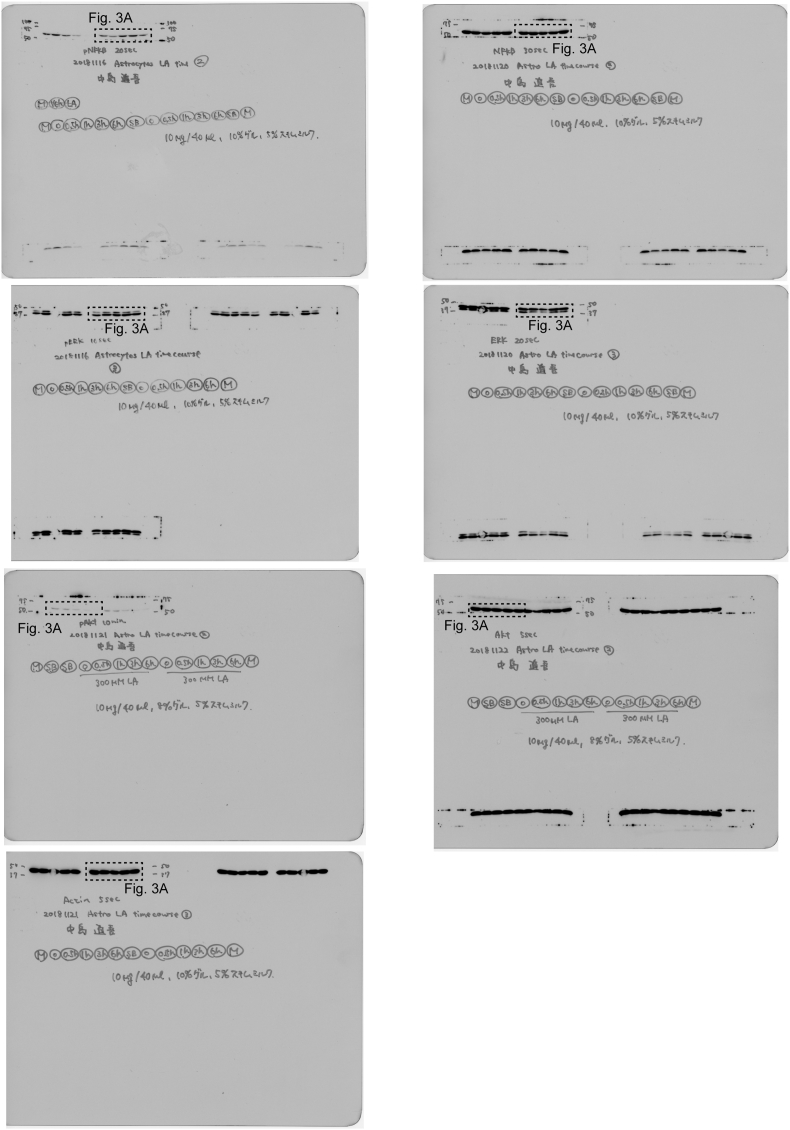


**Supple Ref to Fig4A:**
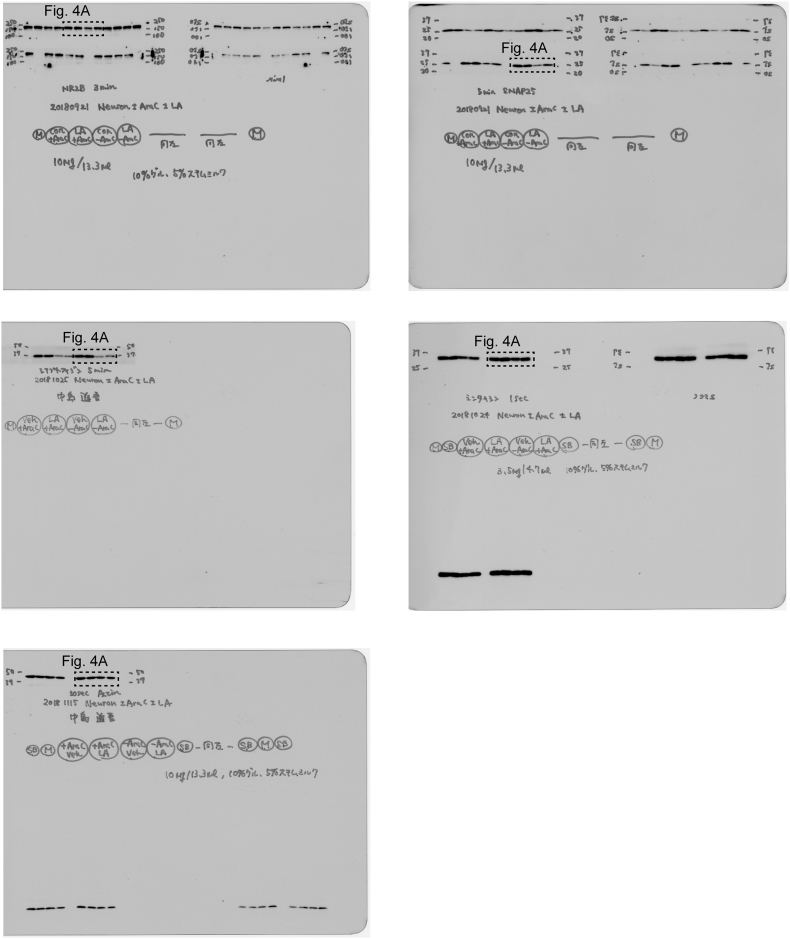


**Supple Ref to Fig4C:**