# Supplementary material for: Lauric acid promotes neuronal maturation mediated by astrocytes in primary cortical cultures
Source: Heliyon. 2020 May 11;6(5):e03892. doi: 10.1016/j.heliyon.2020.e03892 (PMC7218271; doi:10.1016/j.heliyon.2020.e03892)
Supplement: Supplemental Table 1 [file mmc1.docx]

| Supplemental Table 1. Primer set for real-time PCR | | | |  | |  |  |  |  |
| --- | --- | --- | --- | --- | --- | --- | --- | --- | --- |
| Target gene |  | Sequence |  | |  | Target gene |  | Sequence |  |
| *Ngf* | Forward | ctatcctggccactctgaggt | |  | | *Il1b* | Forward | Gccaacaagtggtattctccat | |
|  | Reverse | atctgtgtacggttctgcctgt | |  | |  | Reverse | Gtctttcatcacacaggacagg | |
| *Gdnf* | Forward | actccaatatgcccgaagatta | |  | | *Il6* | Forward | Accaggaaatttgcctattgaa | |
|  | Reverse | cgcttgtttatctggtgacctt | |  | |  | Reverse | Tctgaatgactctggctttgtc | |
| *Igf1* | Forward | caaggggcttttacttcaacaa | |  | | *Il10* | Forward | Gcaggactttaagggttacttgg | |
|  | Reverse | agtacatctccagcctcctcag | |  | |  | Reverse | Atttctgggccatggttctct | |
| *Bdnf* | Forward | cagcacggtaactgacattgat | |  | | *Tgfb* | Forward | Gaagtcacccgcgtgctaat | |
|  | Reverse | gcagagacagacacagaacagg | |  | |  | Reverse | Ccgaatgtctgacgtattgaag | |
| *Egf* | Forward | ctgtcttcctccctgtaactcg | |  | | *Ccl2* | Forward | Ctgtctcagccagatgcagtta | |
|  | Reverse | tttgtcatttgatggtggaatc | |  | |  | Reverse | Gctgctggtgattctcttgtag | |
| *Cntf* | Forward | gattcgttcagacctgactgct | |  | | *Gapdh* | Forward | Ctgcaccaccaactgcttag | |
|  | Reverse | gtcatctcactccaacgatcag | |  | |  | Reverse | Gggccatccacagtcttct | |
| *Tnfa* | Forward | gctccctctcatcagttccat | |  | |  |  |  |  |
|  | Reverse | gcttggtggtttgctacgac | |  | |  |  |  |  |
